# AGE-SPECIFIC TRENDS IN LIMITATIONS TO DAILY ACTIVITIES IN AMERICANS AGED 50 AND OVER BY RACE AND ETHNICITY, 2000–2018

**DOI:** 10.1093/geroni/igae098.2804

**Published:** 2024-12-31

**Authors:** Octavio Bramajo, Neil Mehta, Brandon O’Grady

**Affiliations:** The University of Texas Medical Branch, Galveston, Texas, United States; The University of Texas Medical Branch, Galveston, Texas, United States; The University of Texas Medical Branch, Galveston, Texas, United States

## Abstract

During the first decade of the 2000s the overall prevalence of limitations to daily activities (but not instrumental limitations) has increased in the US. It is not clear if the stagnation in mortality in the last decade, after decades of improvements in life expectancy, was accompanied by stagnation in disability as well or if the pace of increases of disabilities increased. However, it is not clear if specific age-groups drive these trends, and how those differences have manifested by sex, race, and ethnicity since the turn of the century. We used data from the National Interview Health Survey (NHIS), from years 2000 to 2018, and we ran a set of logistic regression models to establish age-specific trends by race for both types of limitations to activities in daily living (stratified by sex, decade and by the age-groups between 50-64,65-74-75-84) to disentangle the effect of time on how those trends developed, controlling by age, education and nativity. From 2000 to 2018 average prevalence of limitations to daily activities and instrumental limitations for adults aged 50 and above has not changed significantly, hovering on similar levels during the whole period. However, we identified an increase of limitations to daily activities and instrumental limitations for the 50-64 age group, with a steep increase during the first decade of the century and a stagnation afterward, mostly driven by Non-Hispanic Blacks and Hispanics, both for males and females. For other age groups, the trend was uneven, and we were unable to distinguish a clear direction.

